# General health, healthcare costs and dental care use of elderly with a natural dentition, implant-retained overdenture or conventional denture: an 8-year cohort of Dutch elderly (aged 75 and over)

**DOI:** 10.1186/s12877-021-02427-z

**Published:** 2021-09-04

**Authors:** Mieke H. Bakker, Arjan Vissink, Gerry M. Raghoebar, Lilian L. Peters, Anita Visser

**Affiliations:** 1 Department of Maxillofacial Surgery, University Medical Center Groningen, University of Groningen, PO Box 30.001, 9700 RB Groningen, the Netherlands; 2 Department of General Practice & Elderly Care Medicine, University Medical Center Groningen, University of Groningen, 9700 RB Groningen, the Netherlands; 3 Epidemiologist/Researcher, Midwifery Science, AVAG, Amsterdam UMC, Vrije Universiteit Amsterdam, Amsterdam Public Health Institute, 1081BT, Amsterdam, the Netherlands; 4 Department of Gerodontology, Center for Dentistry and oral Hygiene, University Medical Center Groningen, University of Groningen, 9700 RB Groningen, the Netherlands

**Keywords:** Community-dwelling elderly, Edentulous, Healthy ageing, Natural dentition, Oral health

## Abstract

**Background:**

Cross-sectional studies have shown that elderly with a natural dentition have better general health than edentulous elderly, but this has not been confirmed in studies with longitudinal design.

**Methods:**

This prospective longitudinal study with a follow-up of 8 years aimed to assess differences in general health, healthcare costs and dental care use between elderly with a natural dentition and edentulous elderly wearing implant-retained or conventional dentures. Based on data of all national insurance claims for dental and medical care from Dutch elderly (aged ≥75 years) general health outcomes (chronic conditions, medication use), healthcare costs and dental care use could be assessed of three groups of elderly, viz. elderly with a natural dentition, elderly with conventional dentures and elderly with implant-retained overdentures.

**Results:**

At baseline (2009), a total of 168,122 elderly could be included (143,199 natural dentition, 18,420 conventional dentures, 6503 implant-retained overdentures). Here we showed that after 8 years follow-up elderly with a natural dentition had more favorable general health outcomes (fewer chronic conditions, less medication use), lower healthcare costs and lower dental costs – but higher dental care use – than edentulous elderly. At baseline the general health of elderly with an implant-retained overdentures resembled the profile of elderly with a natural dentition, but over time their general health problems became comparable to elderly with conventional dentures.

**Conclusions:**

It was concluded that elderly with a natural dentition had significant better health and lower healthcare costs compared to edentulous elderly (with or without dental implants).

**Supplementary Information:**

The online version contains supplementary material available at 10.1186/s12877-021-02427-z.

## Introduction

For decades, the prevalence of edentulism has declined: more and more elderly retain their natural dentition until advanced age. Among elderly ≥75 years old, the prevalence of edentulism in the United States decreased from 67.3% in 1957–1958 to 24.1% in 2009–2012 [1]. European countries showed a similar decrease [2, 3]. There are, however, large differences between countries, as well as differences between rural areas and cities [2]. In Europe, the prevalence of edentulism among adults ≥65 years old ranges from 69% in Albania to 15% in Austria [4, 5].

This decline of edentulism is primarily the result of improved dental care and the changing attitudes towards oral health and dental care over the last four decades [2]. Previous studies have suggested that maintaining a natural dentition is beneficial. Cross-sectional studies have shown that elderly with a natural dentition have better general health [6, 7]. Elderly who retain their teeth until late in life have a higher quality of life and better oral function than edentulous elderly [7–9].

When elderly become edentulous and their masticatory function decreases, this often affects their diet [10, 11]. Hard, fibrous food that is difficult to masticate is replaced by softer food [12, 13], often with higher levels of cholesterol and saturated fats. As a consequence, their nutritional status and subsequently their general health could be at risk, leading to a higher prevalence of obesity and an increased risk for cardiovascular disease [14, 15]. Furthermore, elderly with good masticatory performance have higher scores on general cognition and verbal fluency than elderly with limited masticatory ability [16]. Next, edentulousness can limit social interaction and lead to avoidance of social activities [14]. Considering the above aspects, several researchers have suggested that edentulousness should be viewed as a disability and that it may even be a predictor of various health issues and shortened longevity [17, 18].

Oral function in edentulous patients suffering from ill-fitting dentures and poor oral function can be regained by placing dental implants that retain an overdenture. Elderly with implant-retained overdentures (IODs) show significantly better scores on oral function, denture satisfaction and oral health-related quality of life than elderly with conventional dentures (CDs) [19–22]. This is the major reason that IODs are now considered as the first choice for treatment of edentulous patients with poor oral function [23, 24].

Nearly all research on oral function in edentulous elderly has been performed in cross-sectional settings. Few studies with a long-term follow-up have been published on edentulousness and general health or on comparisons between edentulous elderly and those with a natural dentition. As a result, little is known about whether elderly with a natural dentition have better general health, and thereby lower healthcare costs, over the long term than edentulous elderly wearing implant-retained or conventional dentures. The hypothesis of this study is that elderly with a natural dentition have better general health outcomes, lower healthcare costs and higher dental care use than elderly wearing implant-retained overdentures (IOD) or conventional dentures (CD). Therefore, the aim of this study was to assess differences in general health, healthcare costs and dental care use between elderly with a natural dentition and edentulous elderly wearing implant-retained (IOD) or conventional dentures (CD) over a period of 8 years. Differences in general health (presence of chronic conditions, medication use and prescribed medication, healthcare use), healthcare costs and dental care use between those with natural dentition and edentulous elderly wearing implant-retained or conventional dentures were monitored during this period.

## Materials and methods

This prospective longitudinal cohort study over 8 years was performed in collaboration with Vektis, an organization that warehouses the data on all healthcare declarations in the Netherlands. The cohort of elderly aged ≥75 years old was formed in 2009 and subsequently followed for 7 years (2010–2016).

### Inclusion and exclusion

Formation of the cohort in 2009 was done according to the dental indicators, based on the Dutch uniform dental treatment codes (UPT codes) and oral surgery codes in case of implant placement, as is shown in Table 1. Based on the type of codes, elderly could be grouped in one of three categories: natural dentition, conventional denture or implant-retained overdenture. Elderly participants who changed oral status during this study and elderly who died were then excluded for further research. Fixed implant-retained dentures are rarely seen in the Netherlands, due to the high reimbursements on removable implant-retained overdentures and are therefore not taken into account.

**Table 1:** Indicators used by Vektis for the cohort of elderly ≥75 years in 2009

	Elderly with natural dentition	Elderly with conventional denture	Elderly with implant-retained overdenture
**Dental care**	Received dental care in 2009	Received a removable upper and lower denture in 2009	Received dental implants and an implant-retained removable overdenture in upper and/or lower jaw in 2009
**Dental treatment**	Received one of following treatments: - Endodontic treatment - Tooth extraction/surgery - Fixed dental prosthesis (without implants) - Periodontal surgery - Direct dental restoration	Received one of following treatments: - New complete denture - Relining or rebasing of upper and lower denture	Received the following treatments: - Placement of dental implants - Implant-retained overdenture

During the period 2009–2016, for each year, the following data were collected:

- Visits to medical professionals, defined as dentists, general practitioners (GP), medical specialists (hospital), physiotherapists, mental health practitioners or allied health professional other than a physiotherapist (i.e. dietician).
- Admission to a nursing home. Data from 2012 to 2016 (data from previous years were not available).
- Healthcare costs according to provider: dentist, GP, hospital, pharmacology, physiotherapy, mental health, paramedical care, nursing home.
- Type of medication received: antithrombotics, bisphosphonates, inhalation corticosteroids, antihypertensives, antidepressants. These medication groups are known to have a major effect on the oral environment, such as oral dryness, medication-related osteonecrosis and post-extraction bleeding.
- Total number of medication received: no drugs used, 1–4 drugs or ≥ 5 drugs (polypharmacy) [25]. Only antithrombotics, bisphosphonates, inhalation corticosteroids, antihypertensives and antidepressants were used to determine the number of medication received.
- Medical conditions: asthma, chronic obstructive pulmonary disease (COPD), cancer, high cholesterol, diabetes, cardiac disease, hypertension, kidney disease, Parkinson’s disease, rheumatoid arthritis. The diagnosis was based on prescribed medication according to a pharmacy-based cost group model [26], which means that specific types of medication prescribed in a base year is used as a marker for chronic conditions.

Furthermore, information regarding elderly who died, elderly who changed oral status (for instance: from edentulous to IOD) was included and socioeconomic status (SES) was indexed based on data provided by the Netherlands Institute for Social Research [27]. The following variables were used to determine SES: average income, percentage of citizens with low income, percentage of citizens with low education level and the percentage of unemployed citizens. SES scores were categorized at municipal level into low, middle and high SES.

### Ethical approval

This study was performed in collaboration with Vektis, an organization that collects data on health insurance claims in the Netherlands. As the database generated for this study was anonymous, no ethics approval or consent had to be obtained. The year 2009 was selected as baseline because this was the first year the coverage of the Vektis database for health insurance claims at health insurance companies was > 90% for medical specialties, which provided acceptable insight into healthcare use in the Netherlands. The year 2016 was selected as the final year in this study because Vektis started in 2018 to make this data available for research.

### Statistics

Descriptive statistics were used to report demographic characteristics, chronic conditions, medication use, prescribed medication and healthcare use. At baseline (2009) dental care use (visiting the dentist at least once) was 100% for all groups, as this was an inclusion criterion for 2009. Therefore, the year 2009 was excluded from the analyses for dental care use. For each year, chi-square tests and Fisher’s exact tests were used to analyze differences between elderly with natural dentition, CD and IOD. The Vektis dataset specified the total amount of healthcare costs per profession for the total group. To determine average healthcare costs on the individual level, healthcare costs were divided by the number of individuals who had accessed this type of medical specialty in each year. Statistical differences between groups were determined using Kruskal-Wallis test. SPSS IBM Statistics version 23.0 (SPSS, Chicago, IL, USA) was used for statistical analysis of the results.

Due to the large number of included elderly, almost all differences between groups are statistically significant (*p* < 0.001). This phenomenon is commonly seen in big data studies; even the smallest differences are statistically significant [28]. However, not every significant difference is relevant to daily practice. Therefore, this study did not focus on statistically significant differences at one point, but rather on the trend throughout the period 2009–2016. There was a special interest in figures that varied or increased by ≥5%.

## Results

### Population

At baseline (2009), a total of 168,122 elderly were included. In this population, 82% (*n* = 143,199) elderly had a natural dentition, 14% (*n* = 24,923) had a CD, and 4% (*n* = 6503) had an IOD. The majority of the elderly (85%, *n* = 147,931) were aged between 75 and 85 years. Edentulous elderly with a CD were on average older than those with natural dentition and IODs. At baseline, 19% of elderly ≥85 years were edentulous with a CD, 11% had natural dentition and 6% had IODs. Elderly with a natural dentition had a higher SES on average than edentulous elderly. Age and SES were significantly different between the three subgroups.

Characteristics of the study population are presented in Table 2. A more detailed version of this table is included in Supplementary Data Table 1.

**Table 2:** Changes in the characteristics of Dutch elderly stratified by oral status in 2009 (baseline) as a function of time

	2009 (baseline)			2012			2014			2016		
	ND^a^	CD^b^	IOD^c^	ND	CD	IOD	ND	CD	IOD	ND	CD	IOD
	143,199	18,420	6503	128,100	14,918	5994	113,420	12,241	5438	97,196	9830	4763
**Demographic characteristics**	**n (%)**	**n (%)**	**n (%)**	**n (%)**	**n (%)**	**n (%)**	**n (%)**	**n (%)**	**n (%)**	**n (%)**	**n (%)**	**n (%)**
**Age**												
75–85 years	127,017 (89%)	14,824 (81%)	6090 (94%)	99,627 (78%)	10,130 (68%)	5057 (84%)	76,791 (68%)	7084 (58%)	4042 (74%)	51,795 (53%)	4363 (44%)	2828 (59%)
≥ 85 years	16,182 (11%)	3596 (19%)	413 (6%)	28,473 (22%)	4788 (32%)	937 (16%)	36,629 (32%)	5157 (42%)	1396 (26%)	45,401 (47%)	5466 (56%)	1935 (41%)
**Socioeconomic status**^d^												
Low	34,846 (24%)	5413 (29%)	1784 (28%)	29,291 (23%)	4063 (27%)	1576 (26%)	36,091 (32%)	4479 (37%)	1977 (26%)	29,447 (30%)	3463 (35%)	1668 (35%)
Middle	56,101 (39%)	7658 (42%)	2806 (43%)	50,546 (39%)	6247 (42%)	2649 (44%)	45,941 (40%)	5182 (42%)	2378 (44%)	38,196 (39%)	4102 (42%)	2080 (44%)
High	52,252 (37%)	5349 (29%)	1913 (29%)	48,263 (38%)	4608 (31%)	1769 (30%)	31,388 (28%)	2580 (21%)	1083 (20%)	29,553 (31%)	2265 (23%)	1015 (21%)
**Chronic conditions**												
Asthma	5152 (4%)	815 (4%)	308 (5%)	4715 (4%)	634 (4%)	293 (5%)	4104 (4%)	485 (4%)	258 (5%)	3290 (3%)	368 (4%)	236 (5%)
Cancer	95 (< 1%)	18 (< 1%)	9 (< 1%)	543 (< 1%)	62 (< 1%)	32 (1%)	40 (< 1%)	18 (< 1%)	18 (< 1%)	47 (< 1%)	18 (< 1%)	18 (< 1%)
Cardiac disease	18,914 (13%)	4019 (22%)	882 (14%)	19,557 (15%)	3334 (22%)	1002 (17%)	18,036 (16%)	2790 (23%)	992 (18%)	15,866 (16%)	2257 (23%)	916 (19%)
COPD^e^	5357 (4%)	1373 (8%)	423 (7%)	5254 (4%)	1122 (8%)	450 (8%)	4636 (4%)	906 (7%)	390 (7%)	3805 (4%)	679 (7%)	341 (7%)
Diabetes	12,665 (9%)	2581 (14%)	723 (11%)	11,646 (9%)	2003 (13%)	731 (12%)	9696 (9%)	1546 (13%)	647 (12%)	7703 (8%)	1135 (12%)	558 (12%)
High cholesterol	21,294 (15%)	2425 (13%)	1137 (18%)	20,930 (16%)	2126 (14%)	1124 (19%)	19,242 (17%)	1798 (15%)	1074 (20%)	16,125 (17%)	1439 (15%)	923 (19%)
Hypertension	74,063 (52%)	10,296 (56%)	3339 (51%)	68,236 (53%)	8355 (56%)	3295 (55%)	59,236 (52%)	6720 (55$)	2973 (55%)	48,879 (50%)	5138 (52%)	2575 (54%)
Kidney disease	571 (< 1%)	127 (< 1%)	31 (1%)	572 (< 1%)	108 (< 1%)	34 (< 1%)	506 (< 1%)	79 (1%)	41 (1%)	390 (< 1%)	52 (< 1%)	21 (< 1%)
Parkinson’s disease	1398 (1%)	230 (1%)	83 (1%)	1353 (1%)	170 (1%)	68 (1%)	1156 (1%)	123 (1%)	51 (1%)	946 (1%)	75 (< 1%)	31 (1 < %)
Rheumatoid arthritis	948 (< 1%)	143 (1%)	49 (1%)	1016 (1%)	133 (< 1%)	70 (1%)	797 (1%)	82 (1%)	52 (1%)	652 (1%)	59 (< 1%)	44 (< 1%)
**Medication use**												
0 drugs	35,588 (25%)	3436 (19%)	1570 (24%)	29,103 (23%)	2847 (19%)	1177 (20%)	25,900 (23%)	2484 (20%)	1041 (19%)	22,855 (24%)	2126 (22%)	930 (20%)
1–4 drugs	95,713 (67%)	12,594 (68%)	4391 (68%)	85,895 (67%)	9997 (67%)	4065 (68%)	75,723 (67%)	7981 (65%)	3669 (67%)	64,519 (66%)	6354 (65%)	3215 (68%)
5 or more drugs (polypharmacy)	11,898 (8%)	2390 (13%)	542 (8%)	13,102 (10%)	2074 (14%)	752 (12%)	11,797 (10%)	1776 (15%)	728 (13%)	9822 (10%)	1350 (14%)	618 (13%)
**Prescibed medication**												
Antithrombotics	62,236 (44%)	9498 (52%)	2900 (45%)	61,734 (48%)	8017 (54%)	3037 (51%)	56,648 (50%)	6713 (55%)	2939 (54%)	49,901 (51%)	5409 (55%)	2666 (56%)
Antihypertensives	85,518 (60%)	12,303 (67%)	3794 (58%)	80,343 (63%)	10,149 (68%)	3861 (64%)	71,375 (63%)	8266 (68%)	3556 (65%)	60,884 (63%)	6568 (67%)	3089 (65%)
Antidepressants	12,528 (9%)	2054 (11%)	690 (11%)	12,298 (10%)	1663 (11%)	721 (12%)	11,403 (10%)	1404 (11%)	700 (13%)	9944 (10%)	1144 (12%)	617 (13%)
Bisphosphonates	14,135 (10%)	1866 (10%)	656 (10%)	12,960 (10%)	1533 (10%)	661 (11%)	10,553 (9%)	1177 (10%)	581 (11%)	8057 (8%)	815 (8%)	484 (10%)
Corticosteroids	14,782 (10%)	2713 (15%)	885 (14%)	13,193 (10%)	2049 (14%)	865 (14%)	11,052 (10%)	1555 (13%)	747 (14%)	8806 (9%)	1132 (12%)	617 (13%)
**Healthcare consuption**												
Dental care	143,199 (100%)	18,420 (100%)	6503 (100%)	100,207 (78%)	1429 (10%)	1698 (28%)	81,536 (72%)	1391 (11%)	1578 (29%)	64,833 (67%)	1034 (11%)	1252 (26%)
General practitioner	141,371 (99%)	18,145 (99%)	6442 (99%)	125,705 (98%)	14,394 (97%)	5929 (99%)	109,721 (97%)	11,617 (95%)	5303 (98%)	88,682 (91%)	8703 (89%)	4418 (93%)
Specialist care	128,444 (90%)	16,622 (90%)	6008 (92%)	116,277 (91%)	13,331 (89%)	5586 (93%)	101,835 (90%)	10,841 (89%)	5017 (92%)	87,359 (90%)	8634 (88%)	4362 (92%)
Nursing home	–	–	–	15,110 (12%)	2907 (20%)	546 (9%)	13,249 (12%)	2228 (18%)	528 (10%)	12,754 (13%)	1882 (19%)	570 (12%)
Mental health	5989 (4%)	959 (5%)	246 (4%)	4847 (4%)	583 (4%)	234 (4%)	3661 (3%)	391 (3%)	193 (4%)	2781 (3%)	307 (3%)	146 (3%)
Physiotherapy	12,426 (9%)	1822 (10%)	526 (8%)	9266 (7%)	1084 (7%)	453 (8%)	7557 (7%)	772 (6%)	385 (7%)	6454 (7%)	580 (6%)	347 (7%)
Allied healthcare	6774 (5%)	1149 (6%)	352 (5%)	4361 (3%)	611 (4%)	221 (4%)	7596 (7%)	910 (7%)	403 (7%)	9121 (9%)	1008 (10%)	433 (9%)
**Mortality**	1864 (1%)	1864 (1%)	540 (3%)	6360 (5%)	1269 (9%)	262 (4%)	7091 (6%)	1163 (10%)	287 (5%)	7866 (8%)	1203 (12%)	357 (8%)

^a^*ND* Natural dentition ^b^ *CD*. Conventional denture ^c^ *IOD* Implant-retained overdenture ^d^ Socioeconomic Status determined by average income, percentage of citizens with low income, percentage of with low education level and the percentage of unemployed citizens. SES scores were determined on municipal level, thereby categorizing the low, middle and high SES^27^. ^e^ *COPD* Chronic obstructive pulmonary disease

### General health

**Fig. 1:**
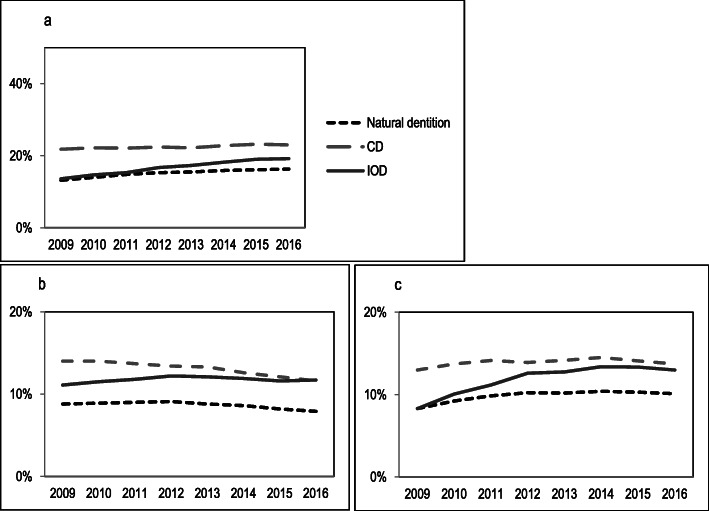
**a** Prevalence of cardiac disease. **b** Prevalence of diabetes. **c** Presence of polypharmacy

An overview of general health and chronic conditions is presented in Table 3. Almost all variables were significantly different between the three groups, except for variables with low prevalence (< 5%), which were cancer, Parkinson’s disease and rheumatoid arthritis. Clinically relevant differences (i.e. difference in prevalence ≥5%) were found for cardiac diseases and diabetes (Fig. 1a and b). Edentulous elderly with CDs were more often diagnosed with these chronic conditions than those with a natural dentition. At baseline, the general health of elderly with an IOD was similar to those with a natural dentition (Fig. 1a and b**)**. During the 7-year follow-up period, the prevalence of cardiac disease and diabetes showed an increase for elderly with IODs, while this figure remained stable for the other groups. At the end of follow-up, the general health of elderly with IODs was similar to the profile of elderly with CDs.

**Table 3:** Healthcare costs (per person) of Dutch elderly in the period 2009–2016

	2009 (baseline)			2012			2014			2016		
	ND^a^	CD^b^	IOD^c^	ND	CD	IOD	ND	CD	IOD	ND	CD	IOD
	***N*** = 143,199	***N*** = 18,420	***N*** = 6503	***N*** = 128,100	***N*** = 14,918	***N*** = 5994	***N*** = 113,420	***N*** = 12,241	***N*** = 5438	***N*** = 97,196	***N*** = 9830	***N*** = 4763
**Healthcare**	**€**	**€**	**€**	**€**	**€**	**€**	**€**	**€**	**€**	**€**	**€**	**€**
Dental care	282	870	3204	251	274	320	262	448	537	255	484	637
General practitioner	156	202	159	159	193	164	193	226	203	181	215	198
Specialist care	2988	3661	3243	3442	3835	3736	3531	3823	3851	3571	3677	3973
Nursing home	–	–	–	31,505	34,285	28,232	42,597	43,407	42,645	48,935	47,361	46,116
Mental health	3345	4246	4395	3026	3363	3615	3726	4236	3377	3697	3593	4817
Physiotherapy	1010	1158	947	1066	1116	1034	1184	1202	1149	1219	1204	1157
Allied healthcare	231	265	203	356	413	337	277	275	271	264	269	267
Pharmacy	1029	1221	1095	962	1132	1063	941	1109	1083	962	1131	1135

^a^*ND* Natural dentition ^b^ *CD* Conventional denture ^c^ *IOD* Implant-retained overdenture

Medication use was highest for elderly with CDs and IODs at baseline (Fig. 1c) and the use of antithrombotics was substantially different between the three groups. The elderly with IODs showed a rapid increase in medication use (especially polypharmacy) and use of antithrombotics; after 8 years, this resulted in a level of medication use comparable to elderly with CDs. Medication used of elderly with a natural dentition and CD increased slowly during this period.

### Dental care and healthcare use

**Fig. 2:**
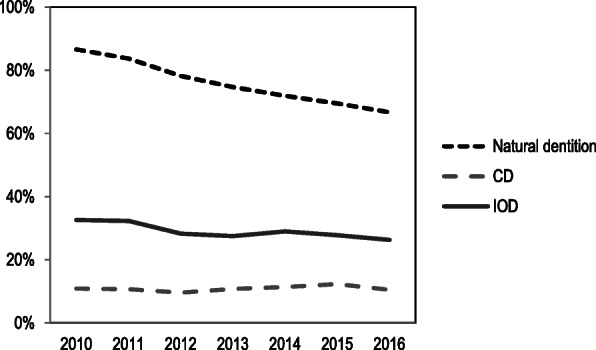
Use of dental care

Dental care use differed between all oral status groups. During follow-up, edentulous elderly had significantly lower dental care use (11% for CD and 26% for IODs) than elderly with natural dentition (67%) (Fig. 2). Such differences were not found for healthcare use at general practitioners and medical specialists. These healthcare providers were visited by around 90% of the elderly from all groups. Nursing home admittance in the period 2012–2016 was highest for elderly with CDs when compared to elderly with a natural dentition and IODs.

### Healthcare costs

The healthcare costs per medical specialty are presented in Table 3 (a more detailed version is presented in Supplementary Data Table 2). Clear differences were found for dental care costs. Elderly with a natural dentition had the lowest and most stable dental care costs throughout follow-up. Edentulous elderly, especially elderly with an IOD, had high costs at baseline when obtaining their new dentures, overdentures and dental implants, followed by a more stable period.

Healthcare costs were highest for the edentulous population. Pharmaceutical costs were lowest for elderly with a natural dentition.

## Discussion

In this cohort study differences in general health, healthcare costs and dental care use between elderly with a natural dentition and edentulous elderly wearing implant-retained overdentures (IODs) or conventional dentures (CDs) were assessed. It was hypothesized that elderly with a natural dentition had better general health outcomes than edentulous elderly. This study showed that edentulous elderly had higher prevalence of general health problems (cardiac disease, diabetes, nursing home admittance), increased medication use (polypharmacy, use of antithrombotics) and higher healthcare costs when compared to elderly with a natural dentition. Within the group of edentulous elderly, those who received an IOD had a general health profile that was comparable to elderly with a natural dentition. Over time, however, their general health problems increased to a level comparable to elderly with CDs.

General health between the groups differed significantly for cardiac disease, diabetes and polypharmacy and the use of antithrombotics. Elderly with CDs showed the highest prevalence of chronic conditions and elderly with a natural dentition showed the lowest prevalence. Elderly with IODs started at the level of elderly with a natural dentition, but during follow-up progressed to the level of edentulous elderly with CDs. The increased prevalence of chronic conditions among the elderly was associated with increased use of polypharmacy and antithrombotics. Consequently, elderly with IODs had both the largest increase in prevalence of chronic conditions and the largest increase in medication use and use of antithrombotics. Previous research shows comparable results regarding the general health outcomes of edentulous elderly with CDs [14]. Diabetes and cardiovascular disease are more prevalent among edentulous elderly [29, 30].

Our finding that the initial prevalence of chronic conditions and medication use in elderly with an IOD is comparable to those with a natural dentition, is in line with the results from the cross-sectional study of Hoeksema et al. [6]. They also reported statistical differences in age between elderly with IODs and CDs, next to the differences in frailty and complex care needs: elderly with IODs were younger and showed better general health. In this study, however, it became clear that over time elderly with an IOD developed a general health profile comparable to elderly with a CD, so their general health deteriorated. Previous research has shown that the lifestyles and diets of edentulous elderly are generally less conducive to health than those with a natural dentition [13]. This is not only due to the loss of oral function, but also to the lower SES of most edentulous elderly [31],which is often related to a less healthy diet. Placing dental implants to retain a lower overdenture will result in improved oral function, but does not automatically lead to a healthier lifestyle [32, 33]. We believe that the elderly who receive an IOD represent a healthier subset of the edentulous elderly at the time they receive dental implants. However, over time their general health profile becomes increasingly similar to the edentulous elderly with CDs, even though their oral function has been improved by placing dental implants. It is likely that the lifestyle accompanying edentulousness may have a negative effect on general health and that this negative effect cannot be prevented by placing dental implants.

Dental care use varied greatly among elderly depending on their oral status. In the period 2010–2016, only 10% of the edentulous elderly visited the dentist after receiving CDs, while 30% of the elderly with IODs continued to visit the dentist. Comparing these results to other European countries, it becomes clear that overall dental attendance and use of preventive treatments of the Netherlands is high and comparable to Sweden, Denmark, Germany and Switzerland [34]. In this study only a small percentage of the elderly with an CD or IOD continued to visit their dentist for routine checkups, although the guidelines for IODs advise annual recall visits to ensure good peri-implant health [35, 36]. In elderly with a natural dentition, there was a decrease in dental care utilization over time as well, although more gradually than among elderly with CDs or IODs. The reason for this is unclear, but it is possible that the interest in oral health diminishes during aging as other health-related problems require more attention. Research in older adults has shown that an oral health problem (e.g. tooth loss) does not substantially influence their subjective oral health [37]. Although regular visits to the dentist decline during aging, visits to the general practitioner and specialist care do not, possibly because they are considered to be more important or more urgent. We would like to suggest that all healthcare providers should stimulate dental care use among elderly and to ask them for oral health complaints as this could result in early detection of poor oral health and prevention of oral pain, discomfort and negative effects on general health outcomes, such a deregulation of diabetes or cardiovascular problems.

Several differences in dental healthcare costs between the three groups were observed. Between 2009 and 2016, the costs for elderly with a natural dentition were the most stable and lowest, while the costs for elderly with dentures (both CD and IOD) were higher and fluctuated more. A possible explanation for this difference is the fact that elderly with conventional dentures and IODs received a new full denture at baseline. Elderly receiving an IOD, which are relatively expensive, incurred especially high costs at baseline. This is well-described in cost-effective studies, showing that IODs are generally 3 to 6 times as expensive as CDs [38, 39]. Their dental costs remain quite high in the period 2010–2016, probably as a result of repair or replacement of their IODs. Throughout the follow-up period, elderly with a natural dentition also had the lowest costs for medication, general practitioner care and specialist care.

In this study elderly aged ≥75 years old were included. Previously, it has been suggested that the definition of old age should be redefined from ≥65 years to ≥75 years, as current elderly are staying robust and active until higher age [40]. This study mainly focuses on general health outcomes. As prevalence of chronic diseases increases with age, it was decided to focus on the oldest proportion of elderly, with more chronic conditions and medication use, rather than including younger (healthier) elderly (aged 65–75 years old).

### Limitations

This study was based entirely on healthcare insurance claims. No formal diagnoses made by doctors or dentists were used. Therefore, elderly participants could only be categorized based on their received dental care by their insurance claims at their insurance companies. As for general health; The Vektis’ database can only register types of medication as used by the elderly. In order to determine presence or absence of a certain type of disease, we can only rely on medication use, also known as pharmacy-based cost group model. Furthermore, elderly who did no claim any dental care costs in the year 2009 could not be identified in the Vektis’ database. As a result, this big data study represents a large part of the Dutch elderly population, but not the entire population. Information on oral health, i.e. peri-implant health, fitting of the dentures, oral pathologies (such as periodontitis or caries), oral function and sufficient functional tooth units was also unavailable. Oral health and oral health problems could therefore not be included. Future studies should aim to start with similar sized groups of elderly with differing oral status, ideally matched by age and SES.

## Conclusions

The general health outcomes of elderly with a natural dentition are better (fewer chronic conditions, less medication use) and more stable than the outcomes of edentulous elderly with an IOD or conventional denture. Elderly with a natural dentition have higher dental care use, but lower healthcare costs than edentulous elderly. The general health of elderly with an IOD initially resembles that of elderly with a natural dentition, but over time their general health declines and becomes comparable to that of elderly with conventional dentures.

## Supplementary Information

**Additional file 1:.** Table 1 Characteristics of Dutch elderly stratified by oral status in the period 2009-2016.

**Additional file 2:.** Table 2 Healthcare costs (per person) of Dutch elderly in the period 2009–2016.

## Data Availability

All data generated or analyzed during this study are included in this published article [and its supplementary information files].

## Abbreviations

CD: Conventional denture
IOD: Implant-retained overdenture
GP: General practitioner
SES: Socioeconomic status
COPD: Chronic obstructive pulmonary disease
