# Supplementary material for: General health, healthcare costs and dental care use of elderly with a natural dentition, implant-retained overdenture or conventional denture: an 8-year cohort of Dutch elderly (aged 75 and over)
Source: BMC Geriatr. 2021 Sep 4;21:477. doi: 10.1186/s12877-021-02427-z (PMC8418734; doi:10.1186/s12877-021-02427-z)
Supplement: Supplementary file 1 — Additional file 1:. Table 1 Characteristics of Dutch elderly stratified by oral status in the period 2009-2016. [file 12877_2021_2427_MOESM1_ESM.docx]

**Table 1 supplementary data: characteristics of Dutch elderly stratified by oral status in the period 2009-2016.**

|  | | **2009** | | | | **2010** | | | | **2011** | | | | **2012** | | | |
| --- | --- | --- | --- | --- | --- | --- | --- | --- | --- | --- | --- | --- | --- | --- | --- | --- | --- |
|  | | **ND^1^** | **CD^2^** | **IOD^3^** | ***p* value^4^** | **ND** | **CD** | **IOD** | ***p* value** | **ND** | **CD** | **IOD** | ***p* value** | **ND** | **CD** | **IOD** | ***p* value** |
|  | | **n (%)** | **n (%)** | **n (%)** |  | **n (%)** | **n (%)** | **n (%)** |  | **n (%)** | **n (%)** | **n (%)** |  | **n (%)** | **n (%)** | **n (%)** |  |
| **DEMOGRAPHIC CHARACTERISTICS** | | **143 199** | **18 420** | **6 503** |  | **140 088** | **17 618** | **6 427** |  | **134 349** | **16 234** | **6 248** |  | **128 100** | **14 918** | **5 994** |  |
| **Age** | |  |  |  |  |  |  |  |  |  |  |  |  |  |  |  |  |
|  | 75-85 | 127 017  (89%) | 14 824  (81%) | 6 090  (94%) | ≤0.001 | 119 528  (85%) | 13 413  (76%) | 5 855  (91%) | ≤0.001 | 109 917  (82%) | 11 716  (72%) | 5 507  (88%) | ≤0.001 | 99 627  (78%) | 10 130  (68%) | 5 057  (84%) | ≤0.001 |
|  | ≥ 85 years | 16 182  (11%) | 3 596  (19%) | 413  (6%) | ≤0.001 | 20 560  (15%) | 4 205  (24%) | 572  (9%) | ≤0.001 | 24 432  (18%) | 4 518  (28%) | 741  (12%) | ≤0.001 | 28 473  (22%) | 4 788  (32%) | 937  (16%) | ≤0.001 |
| **Socioeconomic status^5^** | |  |  |  |  |  |  |  |  |  |  |  |  |  |  |  |  |
|  | Low | 34 846  (24%) | 5 413  (29%) | 1 784  (28%) | ≤0.001 | 34 004  (24%) | 5 189 (29%) | 1 775 (28%) | ≤0.001 | 32 484  (24%) | 4 766 (29%) | 1 725 (28%) | ≤0.001 | 29 291  (23%) | 4 063 (27%) | 1 576 (26%) | ≤0.001 |
|  | Middle | 56 101  (39%) | 7 658  (42%) | 2 806  (43%) | ≤0.001 | 54 924  (39%) | 7 321 (42%) | 2 770 (43%) | ≤0.001 | 52 773  (39%) | 6 768 (42%) | 2 702 (43%) | ≤0.001 | 50 546  (39%) | 6 247 (42%) | 2 649 (44%) | ≤0.001 |
|  | High | 52 252  (37%) | 5 349  (29%) | 1 913  (29%) | ≤0.001 | 51 160  (37%) | 5 108  (29%) | 1 882  (29%) | ≤0.001 | 49 092  (37%) | 4 700 (29%) | 1 821 (29%) | ≤0.001 | 48 263  (38%) | 4 608 (31%) | 1 769 (30%) | ≤0.001 |
| **CHRONIC CONDITIONS** | |  |  |  |  |  |  |  |  |  |  |  |  |  |  |  |  |
|  | Asthma | 5 152  (4%) | 815  (4%) | 308  (5%) | ≤0.001 | 5 076  (4%) | 760  (4%) | 309  (5%) | ≤0.001 | 4 940  (4%) | 680  (4%) | 312  (5%) | ≤0.001 | 4 715  (4%) | 634  (4%) | 293  (5%) | ≤0.001 |
|  | Cancer | 95  (<1%) | 18  (<1%) | 9  (<1%) | 0.04 | 700  (<1%) | 78  (<1%) | 36  (<1%) | 0.45 | 680  (1%) | 69  (<1%) | 34  (<1%) | 0.47 | 543  (<1%) | 62  (<1%) | 32  (1%) | 0.43 |
|  | Cardiac disease | 18 914  (13%) | 4 019  (22%) | 882  (14%) | ≤0.001 | 19 575  (14%) | 3 918  (22%) | 947  (15%) | ≤0.001 | 19 872  (15%) | 3 593  (22%) | 954  (15%) | ≤0.001 | 19 557  (15%) | 3 334  (22%) | 1 002  (17%) | ≤0.001 |
|  | COPD | 5 357  (4%) | 1 373  (8%) | 423  (7%) | ≤0.001 | 5 369  (4%) | 1 299  (7%) | 434  (7%) | ≤0.001 | 5 398  (4%) | 1 241  (8%) | 449  (7%) | ≤0.001 | 5 254  (4%) | 1 122  (8%) | 450  (8%) | ≤0.001 |
|  | Diabetes | 12 665  (9%) | 2 581  (14%) | 723  (11%) | ≤0.001 | 12 518  (9%) | 2 457  (14%) | 738  (11%) | ≤0.001 | 12 139  (9%) | 2 225  (14%) | 739  (12%) | ≤0.001 | 11 646  (9%) | 2 003  (13%) | 731  (12%) | ≤0.001 |
|  | High cholesterol | 21 294  (15%) | 2 425  (13%) | 1 137  (18%) | ≤0.001 | 21 431  (15%) | 2 307  (13%) | 1 176  (18%) | ≤0.001 | 21 466  (16%) | 2 275  (14%) | 1 176  (19%) | ≤0.001 | 20 930  (16%) | 2 126  (14%) | 1 124  (19%) | ≤0.001 |
|  | Hypertension | 74 063  (52%) | 10 296  (56%) | 3 339  (51%) | ≤0.001 | 73 004  (52%) | 9 736  (55%) | 3 372  (52%) | ≤0.001 | 71 148  (53%) | 9 020  (56%) | 3 354  (54%) | ≤0.001 | 68 236  (53.%) | 8 355  (56%) | 3 295  (55%) | ≤0.001 |
|  | Kidney disease | 571  (<1%) | 127  (<1%) | 31  (1%) | ≤0.001 | 614  (<1%) | 133  (1%) | 30  (<1%) | ≤0.001 | 600  (<1%) | 116  (1%) | 42  (1%) | ≤0.001 | 572  (<1%) | 108  (<1%) | 34  (<1%) | ≤0.001 |
|  | Parkinson’s disease | 1 398  (1%) | 230  (1%) | 83  (1%) | ≤0.001 | 1 430  (1%) | 210  (1%) | 79  (1%) | 0.04 | 1 432  (1%) | 199  (1%) | 65  (1%) | 0.30 | 1 353  (1%) | 170  (1%) | 68  (1%) | 0.57 |
|  | Rheumatoid arthritis | 948  (<1%) | 143  (1%) | 49  (1%) | 0.154 | 1 012  (1%) | 145  (1%) | 67  (1%) | 0.01 | 1 012  (1%) | 141  (1%) | 69  (1%) | 0.01 | 1 016  (1%) | 133  (<1%) | 70  (1%) | 0.004 |
| **MEDICATION USE** | |  |  |  |  |  |  |  |  |  |  |  |  |  |  |  |  |
|  | 0 drugs | 35 588  (25%) | 3 436  (19%) | 1 570  (24%) | ≤0.001 | 33 456  (24%) | 3 311  (19%) | 1 394  (22%) | ≤0.001 | 31 039  (23%) | 3 062  (19%) | 1 268  (20%) | ≤0.001 | 29 103  (23%) | 2 847  (19%) | 1 177  (20%) | ≤0.001 |
|  | 1-4 drugs | 95 713  (67%) | 12 594  (68%) | 4 391  (68%) | ≤0.001 | 93 713  (67%) | 11 889  (67%) | 4 386  (68%) | 0.03 | 90 076  (67%) | 10 876  (67%) | 4 282  (69%) | 0.03 | 85 895  (67%) | 9 997  (67%) | 4 065  (68%) | 0.46 |
|  | 5 or more drugs (polypharmacy) | 11 898  (8%) | 2 390  (13%) | 542  (8%) | ≤0.001 | 12 919  (9%) | 2 418  (14%) | 647  (10%) | ≤0.001 | 13 234  (10%) | 2 296  (14%) | 698  (11%) | ≤0.001 | 13 102  (10%) | 2 074  (14%) | 752  (12%) | ≤0.001 |
| **PRESCIBED MEDICATION** | |  |  |  |  |  |  |  |  |  |  |  |  |  |  |  |  |
|  | Antithrombotics | 62 236  (44%) | 9 498  (52%) | 2 900  (45%) | ≤0.001 | 63 412  (45%) | 9 224  (52%) | 3 021  (47%) | ≤0.001 | 63 095  (47%) | 8 646  (53%) | 3 055  (49%) | ≤0.001 | 61 734  (48%) | 8 017  (54%) | 3 037  (51%) | ≤0.001 |
|  | Antihypertensives | 85 518  (60%) | 12 303  (67%) | 3 794  (58%) | ≤0.001 | 85 576  (61%) | 11 896  (68%) | 3 935  (61%) | ≤0.001 | 83 546  (62%) | 11 017  (68%) | 3 926  (63%) | ≤0.001 | 80 343  (63%) | 10 149  (68%) | 3 861  (64%) | ≤0.001 |
|  | Antidepressants | 12 528  (9%) | 2 054  (11%) | 690  (11%) | ≤0.001 | 12 743  (9%) | 1 996  (11%) | 736  (11%) | ≤0.001 | 12 742  (9%) | 1 832  (11%) | 741  (12%) | ≤0.001 | 12 298  (10%) | 1 663  (11%) | 721  (12%) | ≤0.001 |
|  | Bisphosphonates | 14 135  (10%) | 1 866  (10%) | 656  (10%) | 0.48 | 14 177  (10%) | 1 837  (10%) | 701  (11%) | 0.07 | 13 694  (10%) | 1 681  (10%) | 689  (11%) | 0.085 | 12 960  (10%) | 1 533  (10%) | 661  (11%) | 0.07 |
|  | Corticosteroids | 14 782  (10%) | 2 713  (15%) | 885  (14%) | ≤0.001 | 14 493  (10%) | 2 593  (15%) | 902  (14%) | ≤0.001 | 13 930  (10%) | 2 290  (14%) | 879  (14%) | ≤0.001 | 13 193  (10%) | 2 049  (14%) | 865  (14%) | ≤0.001 |
| **HEALTHCARE CONSUPTION** | |  |  |  |  |  |  |  |  |  |  |  |  |  |  |  |  |
|  | Dental care | 143 199  (100%) | 18 420  (100%) | 6 503  (100%) |  | 121 242  (87%) | 1 922  (11%) | 2 098  (33%) | ≤0.001 | 112 486  (84%) | 1 747  (11%) | 2 018  (32%) | ≤0.001 | 100 207  (78%) | 1 429  (10%) | 1 698  (28%) | ≤0.001 |
|  | General practitioner | 141 371  (99%) | 18 145  (99%) | 6 442  (99%) | 0.002 | 136 698  (98%) | 17 037  (97%) | 6 308  (98%) | ≤0.001 | 132 483  (99%) | 15 743  (97%) | 6 206  (99%) | ≤0.001 | 125 705  (98%) | 14 394  (97%) | 5 929  (99%) | ≤0.001 |
|  | Specialist care | 128 444  (90%) | 16 622 (90%) | 6 008  (92%) | ≤0.001 | 126 580  (90%) | 15 868  (90%) | 5 971  (93%) | ≤0.001 | 122 139  (91%) | 14 597  (90%) | 5 803  (93%) | ≤0.001 | 116 277  (91%) | 13 331  (89%) | 5 586  (93%) | ≤0.001 |
|  | Nursing home | - | - | - |  | - |  | - |  | - | - | - |  | 15 110  (12%) | 2 907  (20%) | 546  (9%) | ≤0.001 |
|  | Mental health | 5 989  (4%) | 959  (5%) | 246  (4%) | ≤0.001 | 6 399  (5%) | 966  (5%) | 296  (5%) | ≤0.001 | 6 255  (5%) | 827  (5%) | 305  (5%) | ≤0.001 | 4 847  (4%) | 583  (4%) | 234  (4%) | 0.69 |
|  | Physiotherapy | 12 426  (9%) | 1 822  (10%) | 526  (8%) | ≤0.001 | 13 342  (10%) | 1 854  (11%) | 659  (10%) | ≤0.001 | 12 753  (9%) | 1 583  (10%) | 612  (10%) | ≤0.001 | 9 266  (7%) | 1 084  (7%) | 453  (8%) | 0.64 |
|  | Allied healthcare | 6 774  (5%) | 1 149  (6%) | 352  (5%) | ≤0.001 | 7 734  (6%) | 1 257  (7%) | 416  (6%) | ≤0.001 | 8 226  (6%) | 1 200  (7%) | 441  (7%) | ≤0.001 | 4 361  (3%) | 611  (4%) | 221  (4%) | ≤0.001 |
| **MORTALITY** | | 1 864  (1%) | 540  (3%) | 83  (1%) | ≤0.001 | 4 708  (3%) | 1 257  (7%) | 177  (3%) | ≤0.001 | 5 461  (4%) | 1 253  (8%) | 254  (4%) | ≤0.001 | 6 360  (5%) | 1 269  (9%) | 262  (4%) | ≤0.001 |

|  | | **2013** | | | | **2014** | | | | **2015** | | | | **2016** | | | |
| --- | --- | --- | --- | --- | --- | --- | --- | --- | --- | --- | --- | --- | --- | --- | --- | --- | --- |
|  | | **ND** | **CD** | **IOD** | ***p* value** | **ND** | **CD** | **IOD** | ***p* value** | **ND** | **CD** | **IOD** | ***p* value** | **ND** | **CD** | **IOD** | ***p* value** |
|  | | **n (%)** | **n (%)** | **n (%)** |  | **n (%)** | **n (%)** | **n (%)** |  | **n (%)** | **n (%)** | **n (%)** |  | **n (%)** | **n (%)** | **n (%)** |  |
| **DEMOGRAPHIC CHARACTERISTICS** | | **121 091** | **13 613** | **5 732** |  | **113 420** | **12 241** | **5 438** |  | **105 619** | **11 031** | **5 151** |  | **97 196** | **9 830** | **4 763** |  |
| **Age** | |  |  |  |  |  |  |  |  |  |  |  |  |  |  |  |  |
|  | 75-85 years | 88 531  (73%) | 8 599  (63%) | 4 570  (80%) | ≤0.001 | 76 791  (68%) | 7 084  (58%) | 4 042  (74%) | ≤0.001 | 64 678  (61%) | 5 696  (52%) | 3 513  (68%) | ≤0.001 | 51 795  (53%) | 4 363  (44%) | 2 828  (59%) | ≤0.001 |
|  | ≥ 85 years | 32 560  (27%) | 5 014  (37%) | 1 162  (20%) | ≤0.001 | 36 629  (32%) | 5 157  (42%) | 1 396  (26%) | ≤0.001 | 40 941  (39%) | 5 335  (48%) | 1 638  (32%) | ≤0.001 | 45 401  (47%) | 5 467  (56%) | 1 935  (41%) | ≤0.001 |
| **Socioeconomic status^2^** | |  |  |  |  |  |  |  |  |  |  |  |  |  |  |  |  |
|  | Low | 27 652  (22%) | 3 705 (27%) | 1 490 (26%) | ≤0.001 | 36 091  (32%) | 4 479 (37%) | 1 977 (26%) | ≤0.001 | 32 024  (30%) | 3 891 (35%) | 1 816 (35%) | ≤0.001 | 29 447  (30%) | 3 463 (35%) | 1 668 (35%) | ≤0.001 |
|  | Middle | 47 885  (40%) | 5 724 (42%) | 2 535 (44%) | ≤0.001 | 45 941  (40%) | 5 182 (42%) | 2 378 (44%) | ≤0.001 | 41 591  (40%) | 4 624 (42%) | 2 260 (44%) | ≤0.001 | 38 196  (39%) | 4 102 (42%) | 2 080 (44%) | ≤0.001 |
|  | High | 45 554  (38%) | 4 184 (31%) | 1 707 (30%) | ≤0.001 | 31 388  (28%) | 2 580 (21%) | 1 083 (20%) | ≤0.001 | 32 004  (30%) | 2 516 (23%) | 1 075 (21%) | ≤0.001 | 29 553  (31%) | 2 265 (23%) | 1 015 (21%) | ≤0.001 |
| **CHRONIC CONDITIONS** | |  |  |  |  |  |  |  |  |  |  |  |  |  |  |  |  |
|  | Asthma | 4 408  (4%) | 570  (4%) | 271  (5%) | ≤0.001 | 4 104  (4%) | 485  (4%) | 258  (5%) | ≤0.001 | 3 802  (4%) | 444  (4%) | 235  (5%) | ≤0.001 | 3 290  (3 %) | 368  (4%) | 236  (5%) | ≤0.001 |
|  | Cancer | 482  (<1%) | 50  (<1%) | 33  (1%) | 0.09 | 40  (<1%) | 18  (<1%) | 18  (<1%) | ≤0.001 | 46  (<1%) | *27*  *(<1%)* | 9  (<1%) | ≤0.001 | 47  (<1%) | 18  (<1%) | 18  (<1%) | ≤0.001 |
|  | Cardiac disease | 18 790  (16%) | 3 026  (22%) | 992  (17%) | ≤0.001 | 18 036  (16%) | 2 790  (23%) | 992  (18%) | ≤0.001 | 16 983  (16%) | 2 554  (23%) | 977  (19%) | ≤0.001 | 15 866  (16%) | 2 257  (23%) | 916  (19%) | ≤0.001 |
|  | COPD ^6^ | 4 880  (4%) | 988  (7%) | 415  (7%) | ≤0.001 | 4 636  (4%) | 906  (7%) | 390  (7%) | ≤0.001 | 4 239  (4%) | 808  (7%) | 364  (7%) | ≤0.001 | 3 805  (4%) | 679  (7%) | 341  (7%) | ≤0.001 |
|  | Diabetes | 10 625  (9%) | 1 805  (13%) | 691  (12%) | ≤0.001 | 9 696  (9%) | 1 546  (13%) | 647  (12%) | ≤0.001 | 8 640  (8%) | 1 339  (12%) | 597  (12%) | ≤0.001 | 7 703  (8%) | 1 135  (12%) | 558  (12%) | ≤0.001 |
|  | High cholesterol | 20 270  (17%) | 2 000  (15%) | 1 089  (19%) | ≤0.001 | 19 242  (17%) | 1 798  (15%) | 1 074  (20%) | ≤0.001 | 17 855  (17%) | 1 609  (15%) | 973  (19%) | ≤0.001 | 16 125  (17%) | 1 439  (15%) | 923  (19%) | ≤0.001 |
|  | Hypertension | 63 825  (53%) | 7 503  (55%) | 3 138  (55%) | ≤0.001 | 59 236  (52%) | 6 720  (55$) | 2 973  (55%) | ≤0.001 | 54 081  (51%) | 5 911  (54%) | 2 829  (55%) | ≤0.001 | 48 879  (50%) | 5 138  (52%) | 2 575  (54%) | ≤0.001 |
|  | Kidney disease | 526  (<1%) | 83  (1%) | 34  (1%) | 0.01 | 506  (<1%) | 79  (1%) | *41*  *(1%)* | ≤0.001 | 455  (<1%) | 67  (1%) | 35  (1%) | 0.002 | 390  (<1%) | 52  (<1%) | 21  (<1%) | 0.16 |
|  | Parkinson’s disease | 1 281  (1%) | 155  (1%) | 59  (1%) | 0.66 | 1 156  (1%) | 123  (1%) | *51*  *(1%)* | 0.84 | 1 054  (1%) | 94  (1%) | 38  (1%) | 0.07 | 946  (1%) | 75  (<1%) | 31  (1<%) | 0.01 |
|  | Rheumatoid arthritis | 848  (1%) | 93  (1%) | 62  (1%) | 0.003 | 797  (1%) | 82  (1%) | 52  (1%) | 0.080 | 712  (1%) | 63  (1%) | 54  (1%) | 0.002 | 652  (1%) | 59  (<1%) | 44  (<1%) | 0.07 |
| **MEDICATION USE** | |  |  |  |  |  |  |  |  |  |  |  |  |  |  |  |  |
|  | 0 drugs | 27 701  (23%) | 2 669  (20%) | 1 119  (20%) | ≤0.001 | 25 900  (23%) | 2 484  (20%) | 1 041  (19%) | ≤0.001 | 24 448  (23%) | 2 308  (21%) | 1 014  (20%) | ≤0.001 | 22 855  (24%) | 2 126  (22%) | 930  (20%) | ≤0.001 |
|  | 1-4 drugs | 81 049  (67%) | 9 017  (66%) | 3 882  (68%) | 0.11 | 75 723  (67%) | 7 981  (65%) | 3 669  (67%) | 0.001 | 70 301  (67%) | 7 169  (65%) | 3 450  (67%) | 0.003 | 64 519  (66%) | 6 354  (65%) | 3 215  (68%) | ≤0.001 |
|  | 5 or more drugs (polypharmacy) | 12 341  (10%) | 1 927  (14%) | 731  (13%) | ≤0.001 | 11 797  (10%) | 1 776  (15%) | 728  (13%) | ≤0.001 | 10 870  (10%) | 1 554  (14%) | 687  (13%) | ≤0.001 | 9 822  (10%) | 1 350  (14%) | 618  (13%) | ≤0.001 |
| **PRESCIBED MEDICATION** | |  |  |  |  |  |  |  |  |  |  |  |  |  |  |  |  |
|  | Antithrombotics | 59 423  (49%) | 7 376  (54%) | 3 016  (53%) | ≤0.001 | 56 648  (50%) | 6 713  (55%) | 2 939  (54%) | ≤0.001 | 53 525  (51%) | 6 056  (55%) | 2 849  (55%) | ≤0.001 | 49 901  (51%) | 5 409  (55%) | 2 666  (56%) | ≤0.001 |
|  | Antihypertensives | 76 016  (63%) | 9 220  (68%) | 3 719  (65%) | ≤0.001 | 71 375  (63%) | 8 266  (68%) | 3 556  (65%) | ≤0.001 | 66 275  (63%) | 7 395  (67%) | 3 338  (65%) | ≤0.001 | 60 884  (63%) | 6 568  (67%) | 3 089  (65%) | ≤0.001 |
|  | Antidepressants | 11 812  (10%) | 1 542  (11%) | 695  (12%) | ≤0.001 | 11 403  (10%) | 1 404  (11%) | 700  (13%) | ≤0.001 | 10 707  (10%) | 1 267  (11%) | 667  (13%) | ≤0.001 | 9 944  (10%) | 1 144  (12%) | 617  (13%) | ≤0.001 |
|  | Bisphosphonates | 11 763  (10%) | 1 361  (10%) | 619  (11%) | 0.02 | 10 553  (9%) | 1 177  (10%) | 581  (11%) | 0.002 | 9 306  (9%) | 990  (9%) | 542  (11%) | ≤0.001 | 8 057  (8%) | 815  (8%) | 484  (10%) | ≤0.001 |
|  | Corticosteroids | 12 073  (10%) | 1 844  (14%) | 805  (14%) | ≤0.001 | 11 052  (10%) | 1 555  (13%) | 747  (14%) | ≤0.001 | 10 194  (10%) | 1 356  (12%) | 682  (13%) | ≤0.001 | 8 806  (9%) | 1 132  (12%) | 617  (13%) | ≤0.001 |
| **HEALTHCARE CONSUPTION** | |  |  |  |  |  |  |  |  |  |  |  |  |  |  |  |  |
|  | Dental care | 90 470  (75%) | 1 472  (11%) | 1 574  (28%) | ≤0.001 | 81 536  (72%) | 1 391  (11%) | 1 578  (29%) | ≤0.001 | 73 448  (70%) | 1 360  (12%) | 1 433  (28%) | ≤0.001 | 64 833  (67%) | 1 034  (11%) | 1 252  (26%) | ≤0.001 |
|  | General practitioner | 118 104  (98%) | 13 018  (96%) | 5 630  (98%) | ≤0.001 | 109 721  (97%) | 11 617  (95%) | 5 303  (98%) | ≤0.001 | 97 936  (93%) | 10 007  (91%) | 4 844  (94%) | ≤0.001 | 88 682  (91%) | 8 703  (89%) | 4 418  (93%) | ≤0.001 |
|  | Specialist care | 108 696  (90%) | 12 025  (88%) | 5 299  (92%) | ≤0.001 | 101 835  (90%) | 10 841  (89%) | 5 017  (92%) | ≤0.001 | 94 102  (89%) | 9 711  (88%) | 4 701  (91%) | ≤0.001 | 87 359  (90%) | 8 634  (88%) | 4 362  (92%) | ≤0.001 |
|  | Nursing home | 13 527  (11%) | 2 518  (18%) | 537  (9%) | ≤0.001 | 13 249  (12%) | 2 228  (18%) | 528  (10%) | ≤0.001 | 12 742  (12%) | 1 995  (18%) | 542  (11%) | ≤0.001 | 12 754  (13%) | 1882  (19%) | 570  (12%) | ≤0.001 |
|  | Mental health | 4 702  (4%) | 557  (4%) | 238  (4%) | 0.31 | 3 661  (3%) | 391  (3%) | 193  (4%) | 0.41 | 3 078  (3%) | 321  (3%) | 161  (3%) | 0.68 | 2 781  (3%) | 307  (3%) | 146  (3%) | 0.26 |
|  | Physiotherapy | 8 113  (7%) | 891  (7%) | 419  (7%) | 0.14 | 7 557  (7%) | 772  (6%) | 385  (7%) | 0.14 | 6 964  (7%) | 691  (6%) | 365  (7%) | 0.14 | 6 454  (7%) | 580  (6%) | 347  (7%) | 0.003 |
|  | Allied healthcare | 7 049  (6%) | 910  (7%) | 323  (6%) | ≤0.001 | 7 596  (7%) | 910  (7%) | 403  (7%) | 0.002 | 8 410  (8%) | 965  (9%) | 420  (8%) | 0.02 | 9 121  (9%) | 1 008  (10%) | 433  (9%) | 0.01 |
| **MORTALITY** | | 6 883  (6%) | 1 291  (9%) | 294  (5%) | ≤0.001 | 7 091  (6%) | 1 163  (10%) | 287  (5%) | ≤0.001 | 7 701  (7%) | 1 166  (11%) | 388  (8%) | ≤0.001 | 7 866  (8%) | 1 203  (12%) | 357  (8%) | ≤0.001 |

^1^ ND: Natural dentition

^2^ CD: Conventional denture

^3^ IOD: Implant-supported overdenture

^4^ *p* value: *p* value determined between 3 subgroups

^5^ Socioeconomic Status determined by average income, percentage of citizens with low income, percentage of with low education level and the percentage of unemployed citizens. SES scores were determined on the municipal level, thereby categorizing low, middle and high SES^27^.

^6^ COPD: Chronic obstructive pulmonary disease
