# Supplementary material for: General health, healthcare costs and dental care use of elderly with a natural dentition, implant-retained overdenture or conventional denture: an 8-year cohort of Dutch elderly (aged 75 and over)
Source: BMC Geriatr. 2021 Sep 4;21:477. doi: 10.1186/s12877-021-02427-z (PMC8418734; doi:10.1186/s12877-021-02427-z)
Supplement: Supplementary file 2 — Additional file 2:. Table 2 Healthcare costs (per person) of Dutch elderly in the period 2009–2016. [file 12877_2021_2427_MOESM2_ESM.docx]

**Table 2 supplementary data: healthcare costs (per person) of Dutch elderly in the period 2009-2016.**

|  | | **2009** | | | | **2010** | | | | **2011** | | | | | **2012** | | | |
| --- | --- | --- | --- | --- | --- | --- | --- | --- | --- | --- | --- | --- | --- | --- | --- | --- | --- | --- |
|  | | **ND^1^** | **CD^2^** | **IOD^3^** | ***p* value^4^** | **ND** | **CD** | **IOD** | ***p* value** | **ND** | **CD** | **IOD** | ***p* value** | | **ND** | **CD** | **IOD** | ***p* value** |
|  |  | **N= 143 199** | **18 420** | **6 503** |  | **140 088** | **17 618** | **6 427** |  | **134 349** | **16 234** | **6 248** |  | | **128 100** | **14 918** | **5 994** |  |
| **HEALTHCARE** | | € | € | € |  | € | € | € |  | € | € | € |  | | € | € | € |  |
|  | Dental care | 282 | 870 | 3 204 | ≤0.001 | 245 | 264 | 248 | ≤0.001 | 252 | 240 | 265 | ≤0.001 | | 251 | 274 | 320 | ≤0.001 |
|  | General practitioner | 156 | 202 | 159 | ≤0.001 | 143 | 183 | 149 | ≤0.001 | 153 | 185 | 161 | ≤0.001 | | 159 | 193 | 164 | ≤0.001 |
|  | Specialist care | 2 988 | 3 661 | 3 243 | ≤0.001 | 3 269 | 3 867 | 3 619 | ≤0.001 | 3 421 | 3 942 | 3 875 | ≤0.001 | | 3 442 | 3 835 | 3 736 | ≤0.001 |
|  | Nursing home | - | - | - |  | - | - | - |  | - | - | - |  | | 31 505 | 34 285 | 28 232 | ≤0.001 |
|  | Mental health | 3 345 | 4 246 | 4 395 | ≤0.001 | 3 210 | 3 616 | 4 205 | ≤0.001 | 3 081 | 3 560 | 4 081 | ≤0.001 | | 3 026 | 3 363 | 3 615 | ≤0.001 |
|  | Physiotherapy | 1 010 | 1 158 | 947 | ≤0.001 | 1 035 | 1 089 | 953 | ≤0.001 | 1 072 | 1 102 | 997 | ≤0.001 | | 1 066 | 1 116 | 1 034 | ≤0.001 |
|  | Allied healthcare | 231 | 265 | 203 | ≤0.001 | 247 | 265 | 236 | ≤0.001 | 261 | 269 | 239 | ≤0.001 | | 356 | 413 | 337 | ≤0.001 |
|  | Pharmacy | 1 029 | 1 221 | 1 095 | ≤0.001 | 1 039 | 1 224 | 1 090 | ≤0.001 | 1 031 | 1 199 | 1 123 | ≤0.001 | | 962 | 1 132 | 1 063 | ≤0.001 |
|  | | **2013** | | | | **2014** | | | | **2015** | | | | | **2016** | | | |
|  | | **ND** | **CD** | **IOD** | ***p* value** | **ND** | **CD** | **IOD** | ***p* value** | **ND** | **CD** | **IOD** | | ***p* value** | **ND** | **CD** | **IOD** | ***p* value** |
|  |  | **N= 121 091** | **13 613** | **5 732** |  | **113 420** | **12 241** | **5 438** |  | **105 619** | **11 031** | **5 151** | |  | **97 196** | **9 830** | **4 763** |  |
| **HEALTHCARE** | | € | € | € |  | € | € | € |  | € | € | € | |  | € | € | € |  |
|  | Dental care | 255 | 335 | 369 | ≤0.001 | 262 | 448 | 537 | ≤0.001 | 258 | 425 | 572 | | ≤0.001 | 255 | 484 | 637 | ≤0.001 |
|  | General practitioner | 174 | 206 | 183 | ≤0.001 | 193 | 226 | 203 | ≤0.001 | 169 | 194 | 181 | | ≤0.001 | 181 | 215 | 198 | ≤0.001 |
|  | Specialist care | 3 593 | 3 894 | 3 901 | ≤0.001 | 3 531 | 3 823 | 3 851 | ≤0.001 | 3 443 | 3 635 | 3 652 | | ≤0.001 | 3 571 | 3 677 | 3 973 | ≤0.001 |
|  | Nursing home | 38 051 | 38 587 | 34 861 | ≤0.001 | 42 597 | 43 407 | 42 645 | ≤0.001 | 47337 | 46 256 | 45 089 | | ≤0.001 | 48 935 | 47 361 | 46 116 | ≤0.001 |
|  | Mental health | 3 226 | 3 538 | 4 053 | ≤0.001 | 3 726 | 4 236 | 3 377 | ≤0.001 | 4 035 | 4 973 | 4 690 | | ≤0.001 | 3 697 | 3 593 | 4 817 | ≤0.001 |
|  | Physiotherapy | 1 128 | 1 191 | 1 065 | ≤0.001 | 1 184 | 1 202 | 1 149 | ≤0.001 | 1 200 | 1 164 | 1 158 | | ≤0.001 | 1 219 | 1 204 | 1 157 | ≤0.001 |
|  | Allied healthcare | 275 | 286 | 270 | ≤0.001 | 277 | 275 | 271 | ≤0.001 | 264 | 253 | 269 | | ≤0.001 | 264 | 269 | 267 | ≤0.001 |
|  | Pharmacy | 919 | 1 096 | 1 050 | ≤0.001 | 941 | 1 109 | 1 083 | ≤0.001 | 942 | 1 142 | 1 113 | | ≤0.001 | 962 | 1 131 | 1 135 | ≤0.001 |

^1^ ND: Natural dentition

^2^ CD.: Conventional denture

^3^ IOD: Implant-supported overdenture

^4^ *p* value: *p* value determined between 3 subgroups
